# Reflections on the multi-sectoral response to COVID-19 in Bangladesh’s Rohingya refugee camps

**DOI:** 10.1177/01171968231190331

**Published:** 2023-07-24

**Authors:** George Palattiyil, Md. Tariqul Islam Limon, Md. Fahad Jubayer, Habibur Rahman, Irin Sultana, Mesbha Uddin Ahmed, Dina Sidhva, Harish Nair

**Affiliations:** The University of Edinburgh; Cox’s Bazar District Sadar Hospital; 399806 Sylhet Agricultural University; Upazilla Health and Family Planning Office; Mirzapur Upazilla Health Complex; 54495 Bangladesh Red Crescent Society; 6413 University of the West of Scotland; 3124 The University of Edinburgh

**Keywords:** COVID-19, Rohingya refugees, collaboration, multi-sectoral response, humanitarian aid, Bangladesh

## Abstract

While the COVID-19 pandemic continues to impact people globally, refugees comprise a vulnerable population, particularly those living in densely populated areas. In Bangladesh, Cox’s Bazar is currently home to almost a million Rohingya refugees. Because of the lack of healthcare, sanitation and water, as well as overcrowding, refugees were at high risk of becoming ill during the early phases of the COVID-19 pandemic in 2020. Moreover, superstitions and lack of trust in the healthcare system threaten to put the community at further risk. To prevent tragic consequences, national and international attention and action are required to strengthen the health system for Rohingya refugees. The community will require surveillance and testing, infection prevention and control measures, adequate food supplies, and access to improved healthcare services. This paper calls for a multi-sectoral approach to developing an action plan and implementation strategy to minimize the impact of COVID-19 on this vulnerable population.

## Background

When disaster strikes any part of the world, be it a natural pandemic or economic collapse, people on the margins suffer the most. Displaced refugee populations are likely to lead the list of vulnerable populations. The World Health Organization (WHO) designated the coronavirus pandemic (COVID-19) a global health emergency on 30 January 2020, placing Rohingya refugees in Bangladesh in extremely high danger. In 2017, around a million Rohingya refugees from Myanmar fled to Bangladesh, landing in more than 30 camps in Cox’s Bazar with no access to food, clean water and sanitary facilities (Limon et al., 2020). The camps are extremely crowded (Jubayer et al., 2021). Even before the pandemic, camp residents suffered from severe malnutrition, lack of hygiene and sanitation, heightened sexual violence and significant mental health deterioration (Milton et al., 2017). The first COVID-19 case in the Rohingya camp was discovered on 14 May 2020, when the epidemic initially began (Jubayer et al., 2021). Infections with COVID-19 caused at least 11 Rohingya refugees to pass away, and 507 additional people were infected in Cox’s Bazar camps during the initial breakout phase (Sakib, 2021), which spread to all 34 camps in quick succession (WHO, 2022). Due to a surge in COVID-19 cases in the area in 2021, a 10-day total lockdown was implemented in Cox’s Bazar’s Teknaf Upazila (sub-district), and a week-long equivalent restriction was implemented in five Rohingya camps in the districts of Ukhiya and Teknaf Upazilas (Aljazeera, 2021).

Even in the best circumstances, the presence of many refugees creates difficulties in everyday life for the host community (Elshazly et al., 2019). The possibility of COVID-19 spreading outside of the camps during the epidemic added concerns about the presence of refugees contributing to deforestation and competition in the labor market. In the second half of January 2022, the cumulative number of positive cases was 383, a more than 90 percent increase compared to the start of the month where most were mild cases. The test positivity rate also increased from 0.4 to 12 percent during the same period (UNHCR, 2022). The coronavirus outbreak is likely to impact the psychological well-being of the refugee population significantly. This paper aims to shed light on the risks and effects of COVID-19 on Rohingya refugees in Bangladesh. Refugees require access to healthcare, education and prospects for a better life in addition to food and shelter. This paper explores how the COVID-19 outbreak among the refugee population in Cox’s Bazar would likely affect the health and healthcare systems in the camps that serve as their home.

## Challenges and factors exacerbating the spread of COVID-19

The vaccination program began at the camp level on 10 August 2021, targeting only 30 percent because the priority was the host population (UNHCR, 2022). Controlling the transmission of viruses requires preventive measures such as good hand hygiene, social exclusion, isolation of infected individuals, contact tracing and informational campaigns. But all these preventive measures are challenging to maintain as the refugee camp at Cox’s Bazar is overpopulated with a population density of almost 40,000/km^2^ (Jubayer et al., 2021). Furthermore, findings from a study showed that the Rohingya refugees have abysmal attitudes and practices toward COVID-19. In addition to minimal knowledge about preventive measures, refugees are reluctant to practice these measures, thus complicating the situation further (Jubayer et al., 2022). In times like the COVID-19 pandemic, public health systems are forced to expand beyond regular practice, requiring increased and efficient use of all available resources (Gamage et al., 2016). Responding to the needs of the Rohingyas during this time was difficult due to the lockdowns. Notably, about 88 percent of Rohingyas depend on external aid from United Nations (UN) Agencies and other non-governmental organizations to meet their daily needs (WFP, 2020). Tragically, the repeated lockdowns declared by the government created severe consequences for the Rohingya population to access assistance including health services.

Nearly one-fifth of this population is under five and 8.4 percent are over 50. According to a report in 2018, approximately 14 percent of ever-married women in refugee camps were pregnant (Chowdhury et al., 2018). Global acute malnutrition (GAM) remains a significant issue in this humanitarian context (Kamrujjaman et al., 2021). As a result, much of the population is at risk of viral infection.

In particular, Rohingya women and children face multiple challenges in avoiding COVID-19 infection. Food safety and food security are worsening in the face of the current pandemic. Poor diet and lack of nutrition impair physical condition and decrease the overall quality of life (Yousafzai et al., 2013). Food insecurity and persistent malnutrition are two significant factors that increase the risk of communicable diseases among Rohingya refugees (Chan et al., 2018). Acute malnutrition among children and anemia among women is common (Leidman et al., 2018). The UN has declared that Rohingya children under the age of five typically need fortified food and micronutrient supplementation (OCHA, 2013). Malnourished children are at high risk of COVID-19 (Fore et al., 2020), and significant levels of food insecurity and inadequate sanitation in the camps exacerbate the situation. Many families lack the resources to eat appropriately twice daily (Chan et al., 2018). Waterborne diseases, including diarrhea, cholera, typhoid and hepatitis E, remain endemic in the refugee camps (Chan et al., 2018). To collect drinking water, women and children must gather in a designated location, thus compromising social distancing measures. Chronic malnutrition is likely to suppress their immune systems, increasing the risk of infection (Venter et al., 2020).

Furthermore, a dengue outbreak has recently become more noticeable in this refugee community. Approximately 100,000 dengue cases were reported in 2019, with 164 deaths in Bangladesh (Rahman et al., 2020). Additional diagnostic challenges will likely occur if these camp settings encounter dengue and COVID-19 simultaneously, as the infections share clinical and laboratory characteristics.

The chronic poor health of the Rohingya refugees and the fragile state of the healthcare infrastructure available to the community suggest that a COVID-19 outbreak would have serious consequences. Several communicable and non-communicable diseases are shared among the Rohingyas, including hepatitis A, hepatitis E, cardiovascular disease (CVD), diabetes, asthma and measles (Chan et al., 2018). According to recent studies, people suffering from comorbidities like CVD, diabetes and asthma are at an increased risk of poor outcomes from COVID-19 (Zhou et al., 2020). Fewer data exist on non-communicable diseases in the camps. Still, poor disease management systems, insufficient technical and human resources, and lack of technical personnel in diagnostic laboratories limit the ability to provide adequate care for chronic physical, mental and specialized conditions. There is an urgent need for specialized medical equipment to treat symptomatic COVID-19 patients. Although the health sector has a functioning early warning alert and response system, some cases may be missed due to a lack of routine, mass screening for COVID-19 infection of patients visiting the health facilities, or asymptomatic cases. The Institute of Epidemiologic Disease, Control and Research (IEDCR) facility at Cox’s Bazar Medical College hosted the initial COVID-19 testing, where a shortage of testing kits became a challenge (Banik et al., 2020).

Furthermore, testing was limited to people with a travel history. Due to the possibility of contaminating the local people, Rohingya mobility outside of camps is governed by regulations (Hussain, 2020). Thus, due to these practices, primary detection and surveillance of COVID-19 infection among Rohingya remain challenging.

The Rohingya are disconnected from the outside world due to the lack of internet and telecommunication services in the camps. This inhibits humanitarian actors from effectively disseminating accurate information about the virus; such information is critical for COVID-19 prevention, treatment preparedness and efforts to counter harmful misinformation (Islam et al., 2020). According to a study that surveyed the general population in China during the early stages of the COVID-19 outbreak, the pandemic had moderate to severe psychological effects on more than half of the respondents (Wang et al., 2020). The risk is likely even higher among Rohingya refugees, whose life experiences in Myanmar commonly include trauma that can lead to post-traumatic stress disorders (Kamal et al., 2020). According to a cross-sectional study, the Rohingya face chronic stress associated with daily life in the camps, food scarcity, restrictions on movement and fear of personal safety (Riley et al., 2017). Furthermore, widespread misinformation and misunderstanding concerning COVID-19 feed suspicion and anxiety that prevent people from accessing medical services.

Addressing the lack of health workers to respond to COVID-19 will likely result in improving anti-COVID-19 responses the in the refugee camps. Lacking adequate healthcare facilities in the camps and ensuring sexual and reproductive health services posed a significant challenge even before the COVID-19 pandemic (Truelove et al., 2020). Providing safe facility-based infant delivery, introductory and comprehensive obstetric care services, comprehensive newborn care, contraception and treatment of Human Immunodeficiency Virus (HIV) and other sexually transmitted infections pose further challenges.

During the summer monsoon season (June through mid-October), with storms, cyclones, flash floods and heavy rainfall, further difficulties are created for physicians and health service workers. The overall hygiene and sanitation status in the camps are impacted by environmental catastrophes, leading to a deterioration of latrines, tube wells, shelters and health camps (Elshazly et al., 2019; Islam and Nuzhath, 2018).

The Bangladeshi government has relocated over 19,000 Rohingya refugees from overcrowded refugee camps in the Cox’s Bazar area to the 34-kilometer-offshore island of Bhasan Char (Strangio, 2021). This transfer to a new location with limited facilities is alarming from a public health perspective as it is likely to create problems in managing the COVID-19 infection, causing further public health concerns.

## Perceptions of COVID-19 among the Rohingya

Researchers noted that the community believes the novel coronavirus is dangerous and anticipates that the virus may lead to further problems inside the camps (Lopez-Pena et al., 2020). Primarily, interviewees expressed concern about how the disease is transmitted (Lopez-Pena et al., 2020). Recent studies conducted in the Rohingya camps in Cox’s Bazar (Jubayer et al., 2022; Lopez-Pena et al., 2020) indicate that Rohingya refugees lack knowledge and understanding of respiratory hygiene in social settings, resulting in poor health outcomes. In a recent study, participants reported that fear and stigma related to COVID-19 are increasing, making it difficult for refugees to receive proper medical treatment inside the community. Participants also reported that pregnant women increasingly seek support from traditional Rohingya birth attendants during childbirth due to fear of entering the hospital for delivery and other services (Lopez-Pena et al., 2020). The COVID-19 pandemic exacerbated the fears about the epidemic and furthered the stigma of those who were afflicted due to poor communication, misinformation and paranoia (Limon et al., 2020).

## Responses to COVID-19 in the camp settings

In the Rohingya refugee camps in Cox’s Bazar, refugees aged 55 and over received the second dose of the COVID-19 vaccine during the first half of February 2022. During the six-day vaccine rollout, the vaccine was administered to 33,386 people, 19,919 men and 13,467 women. At the completion of the campaign, 86 percent of this target demographic had received at least one dose of the COVID-19 vaccine, while 77 percent had received both doses (WHO, 2021a). However, these numbers represent only 3.33 percent of the total refugee population. Community Health Workers (CHWs) are continuously conducting active case finding for acute watery diarrhea (AWD), referring people with symptoms, disseminating important health messages and delivering oral rehydration solution (ORS)/Zinc to families reporting symptoms. Furthermore, improved surveillance was initiated through early warning, alert and response system (EWARS) and 22 sentinel testing sites utilized by the World Health Organization (WHO, 2021a).

The Inter Sector Coordination Group (ISCG) in Cox’s Bazar has compiled healthcare capacity statistics and medical training data for the refugee camps. As of 24 June 2020, six Severe Acute Respiratory Infection Isolation and Treatment Centers (SARI-ITC) were operational and could receive severely ill patients. During humanitarian crises, the ISCG is a framework used to promote coordination and cooperation across various humanitarian sectors, organizations and partners involved in response activities. The functional bed capacity of SARI-ITC was 283, with a 27 percent occupancy rate for both the host and Rohingya populations until 2021. Further, as of 24 June 2020, 27 laboratory technicians received training on biosafety and COVID-19 sample collection and transportation, in addition to clinical case management training provided to medical professionals from NGOs.

Additionally, 121 healthcare professionals from health posts, primary healthcare and field hospitals received screening, isolation, infection prevention and control training (ISCG, 2020a). Twelve SARI-ITC were subsequently established and patients in need of intensive care or high-dependency units were referred to the Sadar Hospital in Cox’s Bazar (WHO, 2020). Partner organizations in the water, sanitation and hygiene (WASH) sector gave soap to 28,090 families and family hygiene kits to 4,057 homes in the camps. Additionally, 10,803 handwashing stations were installed, including 4,737 hands-free handwashing machines for use in locations that lack running water (UNHCR, 2020).

A total of 1.6 million reusable cloth masks were distributed to all Rohingya refugees over the age of five living in all 34 camps in Bangladesh as part of the most recent collaboration between aid organizations to help the government of Bangladesh stop the spread of COVID-19 and offer livelihood opportunities to local communities. In addition, the United Nations International Children's Emergency Fund (UNICEF) built a 200-bed COVID-19 Isolation and Treatment Center for patients from Bangladeshi and Rohingya communities as part of the Health Sector’s overall objective to increase bed capacity throughout the district (ISCG, 2021).

The Field Laboratory of the Institute of Epidemiology, Disease Control and Research (IEDCR) at Cox’s Bazar Medical College received support from WHO in the form of staff, tools, consumables, technical and operational assistance. In Cox’s Bazar and the nearby Bandarban area, encompassing both the local population and Rohingya refugees, 131,522 COVID-19 Reverse Transcription-Polymerase Chain Reaction (RT-PCR) tests were performed from early April 2020 to 23 May 2021. Additionally, 2,931 cases were confirmed through COVID-19 awareness sessions (ISCG, 2020b). Systems to deliver food supplies to refugees' homes and the installation of hygiene and sanitation facilities outside the Rohingya community have also been upgraded. Interestingly, it was reported that 1,013 of 42,483 testing samples showed a 2.38 percent positivity rate and a 1.58 percent case fatality rate (16 deaths). In contrast, it was 1.08 percent in the host community (95 deaths) (WHO, 2021b). However, the number has increased by three folds to 3,319 confirmed cases out of 83,207 samples tested with a four percent positivity rate and a one percent case fatality rate within a short six-month period among the Rohingya refugees (WHO, 2022). This shows a sustained increase in cases among Rohingya refugees within the last six months.

## Discussion and recommendations

Rohingya refugees had experienced great suffering before and during their flight to Bangladesh, which intensified during the COVID-19 outbreak. It was challenging to implement social segregation, isolation, contact tracing and enhance cleanliness and sanitation because of the unstable circumstances and overpopulation in the camps (Raju and Ayeb-Karlsson, 2020), which posed formidable public health challenges. If some headway is to be achieved, partnering with influential local religious, community, family and group leaders is crucial. This approach has been shown to have promising results elsewhere (Kabir et al., 2010; Nawab et al., 2006).

## Recommendations

To successfully deal with the ongoing COVID-19 pandemic in Bangladesh, any coherent response must include programs for Rohingya refugees. Disregarding refugees or migrants from national and international approaches violates the ethics that support public health (Orcutt et al., 2020). While it is acknowledged that some government actions have occurred, these have been piecemeal. We put forward the following recommendations to promote the health and well-being of Rohingya as the pandemic approaches its third year.

1. Vaccination must be ensured for the entire Rohingya refugee community, including the young, adolescents and children. Pregnant women may be prioritized.
2. Epidemiological risk assessments must be completed to develop case management protocols and form a rapid response team (Kluge et al., 2020). A skilled epidemiology team should be formed to examine the public health response to COVID-19 in the camps. The team will ensure case detection, contact identification and contact tracing, and coordinate and implement camp-level public health interventions.
3. Soap and facemasks should be distributed to the Rohingya community, ideally reusable. The protocol for using soapy water developed by the International Center for Diarrheal Disease Research, Bangladesh (ICDDR) and approved by the WHO, should be introduced to the community. As reusable facemasks are effective against the COVID-19 virus, government and non-governmental organizations (NGOs) can train the Rohingya refugees, especially women, to produce and distribute such types of masks inside the camps, which can act as non-pharmaceutical intervention (NPI). One of the NGOs, in this instance, is training Rohingya women inside camps to make wearable masks (World Vision International, 2020).
4. Food fortification for Rohingya women and children is critical to boost their immunity against COVID-19 and to help prevent malnutrition and nutritional disorders common in the community. To achieve this goal, local governments, NGOs and food industries may come forward to pilot a food fortification program that can later be scaled up.
5. Electronic billboards and posters with accurate and actionable information about COVID-19 can be posted inside the camps, preferably in the Rohingya language. Employing extension workers would be appropriate for completing these tasks. As previously stated, including camp residents in these activities would be ideal. In addition, short films in the Rohingya language on COVID-19 prevention can be displayed in the waiting rooms of health facilities.
6. Separate COVID-19 testing facilities should be established immediately. There must be a provision for maternity care in each center. All healthcare professionals need to receive proper training in infection, prevention and control (IPC) of severe acute respiratory infection in order to deliver the best possible care.
7. The entire camp or specific areas can be marked and separated into green and red zones depending on the exposure and infectivity of the population. Guidelines developed by Favas (2020) can be highly effective in implementing this.
8. Special consideration should be given to refugees aged over 45 years. Measures should include increased social distancing, awareness raising and regular doorstep follow-ups by community health workers to reduce older camp residents' risk of exposure to the virus. A well-planned vaccination program for all camp residents should begin as soon as possible. In the case of receiving vaccines, priority should be given to those aged above 45 and those having other communicable and non-communicable diseases.
9. Distribution of facemasks and hand sanitizers to all camp residents should be combined with the current food distribution system to reduce the risk of infection in the community. “No mask, no service” provisions can be initiated at all service delivery points, including health facilities and food distribution centers.
10. Roadside handwashing stations can be installed in various areas to encourage hand hygiene.
11. Any future relocation should be done gradually and in stages, only in areas with adequate and structured medical facilities to prevent infection. This is particularly important during seasons of heavy rainfall that can lead to disasters. Forcibly relocating refugees during these precarious seasons is likely to exacerbate the challenges faced by refugees.
